# Nontyphoidal *Salmonella* Infection, Guangdong Province, China, 2012^1^

**DOI:** 10.3201/eid2204.151372

**Published:** 2016-04

**Authors:** Xi Huang, Qiong Huang, Zhongjun Dun, Wei Huang, Shuyu Wu, Junhua Liang, Xiaoling Deng, Yonghui Zhang

**Affiliations:** Guangdong Provincial Center for Disease Control and Prevention, Guangzhou, China (X. Huang, Q. Huang, J. Liang, X. Deng, Y. Zhang);; Guangdong Provincial Institute of Public Health, Guangzhou (Z. Dun);; Liwan District Center for Disease Control and Prevention, Guangzhou (W. Huang);; Centers for Disease Control and Prevention, Atlanta, Georgia, USA (S. Wu)

**Keywords:** Nontyphoidal Salmonella infection, Salmonella, epidemiology, burden of illness pyramid model, bacteria, Guangdong Province, China, foodborne illness

## Abstract

We used active and passive surveillance to estimate nontyphoidal *Salmonella* (NTS) infection during 2012 in Guangdong Province, China. Under passive surveillance, for every reported NTS infection, an estimated 414.8 cases occurred annually. Under active surveillance, an estimated 35.8 cases occurred. Active surveillance provides remarkable advantages in incidence estimate.

Gastrointestinal illness or diarrhea caused by foodborne pathogens, such as nontyphoidal *Salmonella* (NTS), is a global public health concern (*1*). Many countries (e.g., the United States, England and Wales, Australia, Canada, Jordan, and Japan) have estimated the incidence of gastrointestinal illness caused by specific pathogens (*2*–*8*). However, in China, information is limited about the incidence of specific foodborne pathogens.

In 2003, China initiated a national, internet-based disease reporting system called the National Notifiable Disease Reporting System (NNDRS). This system legally requires routine reporting from all medical institutions and public health units of a list of infectious diseases. In this system, diarrheal pathogens other than *Vibiro cholerae* and *Shigella dysenteriae*, such as *Salmonella* spp., *Escherichia coli*, and *Listeria* spp., are reported as “other infectious diarrhea”; information about etiology is provided as an additional comment (*9*). Because NNDRS is passive, few reports include laboratory confirmation. According to previous data from passive surveillance, <1,000 NTS cases were reported in Guangdong Province annually since 2009, representing only a small proportion of actual infections.

In 2009, Guangdong Provincial Center for Disease Control and Prevention (Guangdong CDC) established laboratory-based active surveillance for NTS infection. In 2012, this system covered more than half of the Guangdong Province prefectures, capturing 61.5% of the population served by 27 sentinel hospitals (21 general hospitals and 6 specialized hospitals, including pediatric and gynecologic). In the surveillance system, patients with >3 loose stools in a 24-hour period plus fever, vomiting, or abdominal pain who visited the sentinel hospitals were enrolled as cases, and fecal samples were collected. The sentinel hospitals were required to forward *Salmonella* isolates to Guangdong CDC, along with epidemiologic data, for analysis. Culture-confirmed cases were then reported to NNDRS with pathogen information. Based on the pyramid model of burden of illness, we used data from active and passive surveillance to estimate NTS infection and to clarify the advantages and disadvantages of each system (*2*,*7*,*10*).

## The Study

The estimation requires multiple steps. First, a person must have symptoms severe enough for medical care (multiplier 1). Second, the physician must collect patients’ specimens (multiplier 2) and forward them for testing by bacterial culture (multiplier 3). Third, the sample test result must be positive (multiplier 4), and the confirmed case must be reported (multiplier 5) (*2*,*7*,*8*).

To obtain multiplier 1, we conducted a 12-month population-based household survey during March 1, 2012–February 28, 2013 (approved by the Ethics Committee of Guangdong CDC). Respondents were randomly selected from 4 districts in western, eastern, and central Guangdong Province. The case definition was the same as that for active surveillance. We used a standard questionnaire to collect information about diarrhea in the previous 4 weeks. The incidence rate of diarrhea was 0.1081 (95% CI 0.1004–0.1158) episodes/person-year; 38.6% of the household survey respondents with diarrhea sought medical care. Multipliers 2 and 3 were based on data from sentinel hospitals and comprised the overall number of diarrhea cases, samples collected, and samples submitted for culture during the year. A total of 75,583 (45.3%) samples of 166,729 registered diarrhea cases in the sentinel hospitals were collected, of which 22,577 (29.9%) were tested. Laboratories of sentinel hospitals cultured samples for *Salmonella* in accordance with standard protocol provided by the national reference laboratory by using MacConkey agar as plating medium. According to a proficiency testing program, the *Salmonella* isolation sensitivity rate of these laboratories was 87.5% (multiplier 4). The numbers of *Salmonella* isolates identified and reported to NNDRS as NTS infectious diarrhea by all sentinel hospitals yielded the proportion of cases reported (648/1,061, 61.1%) (multiplier 5). We estimated the number of infections using the above 5 multipliers. Thus, active surveillance for each reported NTS infection identified 35.8 cases. We also analyzed multipliers of specialized hospitals (Table 1).

**Table 1:** Active and passive surveillance multipliers used to determine the incidence of nontyphoidal *Salmonella* infections, Guangdong Province, China, 2012*

Surveillance steps	Active surveillance				Passive surveillance, by age group, y					
	Overall	General hospitals	Specialized hospitals		Overall	0–4	5–24	25–44	45–64	>65
Multiplier 1: Patient seeks medical care	2.59	2.59	2.59		2.59	1.25	5.0	8.0	2.0	3.0
Multiplier 2: Physician obtains samples	2.21	2.01	2.31		2.21	2.21	2.21	2.21	2.21	2.21
Multiplier 3: Samples tested for *Salmonella*	3.35	2.45	6.73		3.35	3.35	3.35	3.35	3.35	3.35
Multiplier 4: Positive laboratory test result	1.14	1.14	1.14		2.07	2.07	2.07	2.07	2.07	2.07
Multiplier 5: Confirmed cases reported	1.64	1.60	1.82		10.45	10.45	10.45	10.45	10.45	10.45
Overall	35.8	23.3	83.7		414.8	200.1	800.6	1,280.9	320.2	480.4

*Incidence is cases per 100,000 persons.

In the population survey–based passive surveillance system, multiplier 1 was the same as for active surveillance. According to a comparison with samples from the submission proportion in a survey of physician-diagnosed diarrhea in Guangdong Province during 2009 (Mann-Whitney test, p = 0.246) (*11*), and a comparison between medical institutions that charged and did not charge for testing (Kolmogorov-Smirnov test, p = 0.837), the proportion of samples submitted and tested from active surveillance were also used as estimates of passive surveillance. The average test sensitivity of sentinel laboratories before active surveillance began was used as an estimate of all medical institutions (i.e., the sensitivity of passive surveillance [48.2%]). Using numbers of *Salmonella* isolates in Guangdong Province from laboratory data, and number of reported NTS cases by all medical institutions, we determined the proportion of reported NTS was 9.6% (991/10,360). Thus, for each reported NTS case under passive surveillance, 414.8 cases actually occurred. Multipliers of 5 age groups also were presented (Table 1).

To generate a more robust estimate, we conducted uncertainty and sensitivity analyses (online Technical Appendix, http://wwwnc.cdc.gov/EID/article/22/4/15-1372-Techapp1.pdf) on passive surveillance data using Monte Carlo simulation (@Risk 6.0; Palisade, Ithaca, NY, USA) (*12*). We used β distribution to describe the uncertainty of proportions and negative binominal distribution to estimate the number of cases. The sensitivity analysis helped determine factors that provide higher uncertainty in the estimate.

The uncertainty analysis model predicted a 411.9 (95% CI 308.4–592.7) overall multiplier and estimated that 408,499 (95% CI 302,899–591,901) *Salmonella* cases occurred per year when the overall multiplier was applied to the 991 reported NTS cases, resulting in 391.6 (95% CI 290.3–567.4) cases/100,000 persons in 2012. Incidence for 5 age groups was also estimated (Table 2). The rank correlation of various factors in the model showed that patients seeking medical care provided the highest uncertainty in the overall estimate (influence rate 96%) (Figure).

**Table 2:** Results of Monte Carlo simulation characterizing the uncertainty about the number of cases and the passive surveillance multipliers and the annual reported and estimated cases and incidence of nontyphoidal *Salmonella* infections, Guangdong Province, China, 2012

Age group, y	Annual no. cases, median (95% CI)	Multipliers, median (95% CI)	No. cases			Annual incidence, cases/100,000	
			Reported	Estimated (95% CI)		Reported	Estimated (95% CI)
0–4	87,195 (68,367–138,725)	209.6 (168.5–332.1)	415	87,195 (68,367–138,725)		6.13	1,287.9 (1,009.8–2,049.0)
5–24	222,558 (90,222–1,376,278)	605.3 (248.9–3720.3)	367	222,558 (90,222–1,376,278)		0.90	547.7 (222.0–3387.1)
25–44	119,409 (49,292–488,534)	1038.3 (436.4–4250.9)	115	119,409 (49,292–488,534)		0.32	333.4 (137.6–1,364.1)
45–64	18,577 (11,400–36,234)	320.2 (216.8–603.2)	58	18,577 (11,400–36,234)		0.40	126.6 (77.7–247.0)
>65	16,421 (8,351–42,273)	457.1 (259.7–1156.5)	36	16,421 (8,351–42,273)		0.56	255.1 (129.7–656.8)
Overall	408,499 (302,899–591,901)	411.9 (308.4–592.7)	991	408,499 (302,899–591,901)		0.95	391.6 (290.3–567.4)

**Figure:**
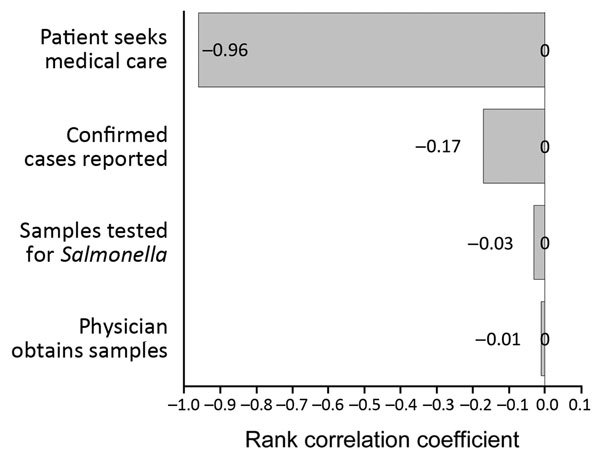
Rank correlations for the total number of nontyphoidal *Salmonella* cases in the population (tornado diagram), Guangdong Province, China, 2012.

## Conclusions

Our estimated NTS incidence was lower than the incidence in China as determined from a literature review (626.5 cases/100,000 persons) (*13*) but close to that in the United States (352.1 cases/100,000 persons) (*3*). However, incidences for persons <5 years of age and 5–24 years of age in our study were higher than those for persons in China and the United States, highlighting that *Salmonella* represents a major health problem in Guangdong Province, especially among younger persons. Our estimated active surveillance rate (35.8) of NTS infections per reported case is similar to estimates in the United States (38.6 and 39) (*2*,*10*) but different from those for England (3.2), Jordan (278), and Japan (63) (*7*,*8*,*14*). Such differences might be due to differences in methods used and to actual differences in *Salmonella* infections.

With fewer missing cases and less underestimation, active surveillance has lower overall multipliers than passive surveillance, indicating smaller surveillance artifacts and more accurate incidence estimate and presents remarkable advantages over passive surveillance. The estimate for active surveillance also showed that if we seek to reduce uncertainty in the overall estimate, we should first focus on encouraging patients to seek medical care.

Our study provides policymakers in China with a reference for the importance of *Salmonella* incidence and calls for balanced surveillance on both foodborne infections and foods and enlarging active surveillance scales. More surveillance guidelines need to be developed to help physicians identify timing of sampling, tests, and performance. Laws requiring reporting of foodborne diseases and pathogens need to be enacted to increase quantity and quality of reporting. The result suggests that to increase care seeking and sample submission, government health insurance schemes should be further developed to cover diagnostic tests and treatments of diseases of public health significance.

Technical Appendix Uncertainty and sensitivity analysis and study limitations for a study of nontyphoidal *Salmonella* infection, Guangdong Province, China, 2012.
