# Supplementary material for: Nontyphoidal Salmonella Infection, Guangdong Province, China, 2012
Source: Emerg Infect Dis. 2016 Apr;22(4):726–9. doi: 10.3201/eid2204.151372 (PMC4806953; doi:10.3201/eid2204.151372)
Supplement: Technical Appendix — Uncertainty and sensitivity analysis and study limitations for a study of nontyphoidal Salmonella infection, Guangdong Province, China, 2012. [file 15-1372-Techapp-s1.pdf]

Article DOI: http://dx.doi.org/10.32032/eid2204.151372

Nontyphoidal *Salmonella* Infection, Guangdong Province, China, 2012

Technical Appendix

Uncertainty and sensitivity analysis

From data of a population-based survey, we found that 22 (38.6%) of 57 persons with diarrhea reported seeking medical care. From surveillance data, 75,583 of 166,729 registered persons with diarrhea sought medical care in the surveillance hospitals submitting fecal samples; 22,577 of submitted samples tested positive for *Salmonella*; the laboratory test sensitivity was 48.20%; and of the total 10,360 *Salmonella* isolates, 991 were reported in the National Notifiable Disease Reporting System as nontyphoidal *Salmonella*.

In the uncertainty analysis on passive surveillance data using Monte Carlo simulation (*1*), the β distribution was used to describe uncertainty about proportions (functions and parameters are presented in Technical Appendix Table 1). The negative binomial distribution was used in stepwise fashion to add the number of cases that are missed by the surveillance system due to surveillance artifacts; thus, the total number of cases (N) = no. of cases reported (n) + negative binomial (n, p), p = $p_1 \times p_2 \times p_3 \times p_4 \times p_5$ (p is joint probability).

In the sensitivity analysis, we used variance rates of median and 95% CI of annual cases to describe the influence of the joint probability p (p = $p_1 \times p_2 \times p_3 \times p_4 \times p_5$) on the annual cases estimate (Technical Appendix Table 2). The result indicated that the median of annual cases had more stable results (ranging from –25% to 25%), when the variance rate of joint probability p ranging from –20% to 30%, but had poor stability (30%~45%) when the variance rate of p ranging from –30% to –25%.

The uncertainty of the overall estimate that the input variables (p_1, p_2, p_3, p_4, p_5) provided was described by the rank correlations and presented in descending order in the tornado diagram (Figure). As the figure showed, 4 variables, including patients seeking medical care, confirmed

cases reported, samples tested for *Salmonella*, and physician-obtained samples, provided negative influence on overall estimate of annual cases, means the higher these proportions are, the lower the multipliers, as well as the lower the uncertainty of the annual cases estimate. With the highest influence rate (96%), patients seeking medical care provided the highest uncertainty, and the confirmed cases reported came second (influence rate 17%); samples tested for *Salmonella* and physician-obtained samples provided lower uncertainties, with influence rate <3%.

Study Limitations

Our study has several limitations. First, the samples of the population survey were not totally the same as active surveillance. Second, only a small number of hospitals were included in active surveillance; because the numbers of each surveillance steps were collected separately, the duplicated and invalid samples could not be corrected. Third, we assumed that the behaviors of care seeking were the same between persons with nontyphoidal*Salmonella*and persons with diarrhea. However, salmonellosis may have severer symptoms and longer durations with greater needs of medical care. Forth, some proportions from active surveillance were used as estimate of passive surveillance, as in active surveillance physicians and laboratorians take more initiative in treatment, testing, and reporting, it might have underestimated the real incidence. Finally, only the available data were used to estimate the average test sensitivity in passive surveillance but no specific surveys.

Reference

1. Powell M, Ebel E, Schlosser W. Considering uncertainty in comparing the burden of illness due to foodborne microbial pathogens. Int J Food Microbiol. 2001;69:209–15. PubMed http://dx.doi.org/10.1016/S0168-1605(01)00495-0\\

Technical Appendix Table 1. Uncertainty analysis on passive surveillance data, functions, and parameters, nontyphoidal *Salmonella* Infection, Guangdong Province, China, 2012

	β Distribution (proportion)		
Surveillance steps	Functions	Parameter 1	Parameter 2
Confirmed cases reported	$P_5 \approx \beta$ (991 + 1, 10360 – 991 + 1)	992	9370
Laboratory test sensitivity	$P_4 = 0.482$	0.482	0.482
Samples tested for *Salmonella*	$P_3 \approx \beta$ (22577 + 1, 75583 – 22577 + 1)	22578	53007
Physician obtains samples	$P_2 \approx \beta$ (75583 + 1, 166729 – 75583 + 1)	75584	91147
Patient seeks medical care	$P_1 \approx \beta$ (22 + 1, 57 – 22 + 1)	23	36
Overall (no. cases)	No. cases reported (*n*) + Negbin (*n, p*) p = $p_1 \times p_2 \times p_3 \times p_4 \times p_5$		

Technical Appendix Table 2. Influence of the joint probability on the annual estimate of cases, nontyphoidal *Salmonella* Infection, Guangdong Province, China, 2012

	Annual no. cases		
Variance rate of joint probability, %	Median	95% CI	Variance rate of median, %
**+30**	**314,378**	**233,396–456,756**	**–23.0**
**+25**	**326,783**	**243,171–472,064**	**–20.0**
**+20**	**340,153**	**253,116–494,260**	**–16.7**
**+15**	**356,009**	**263,385–515,700**	**–12.8**
**+10**	**371,944**	**275,867–537,890**	**–9.0**
**+5**	**388,447**	**289,986–567,123**	**–4.9**
**–5**	**430,446**	**317,977–621,347**	**+6.0**
**–10**	**453,821**	**338,154–658,548**	**+11.1**
**–15**	**480,674**	**356,927–696,189**	**+17.7**
**–20**	**511,328**	**378,014–742,446**	**+25.1**
–25*	544,296	402,476–789,619	+33.2
–30	584,342	431,695–844,239	+43.0
Overall	408,499	302,899–591,901	–

*Bold indicates the more stable results (with variance rate of median of –25% to 25%).
